# Genetic population structure of invasive raccoons (*Procyon lotor*) in Hokkaido, Japan: Unique phenomenon caused by pet escape or abandonment

**DOI:** 10.1038/s41598-020-64526-y

**Published:** 2020-05-15

**Authors:** Minami W. Okuyama, Michito Shimozuru, Mariko Nakai, Emi Yamaguchi, Kei Fujii, Ken-ichiro Shimada, Tohru Ikeda, Toshio Tsubota

**Affiliations:** 1 Research Promotion Institute, Oita University, Oita, 879-5593 Japan; 2 Laboratory of Wildlife Biology and Medicine, Faculty of Veterinary Medicine, Hokkaido University, Hokkaido, 060-0818 Japan; 3 Department of Regional Science, Division of Human Sciences, Faculty of Humanities and Human Sciences, Hokkaido University, Hokkaido, 060-0810 Japan; 4 Obihiro University of Agriculture and Veterinary Medicine, Hokkaido, 080-8555 Japan; 5 Animal Research Center, Hokkaido Research Organization, Hokkaido, 081-0038 Japan

**Keywords:** Population genetics, Invasive species

## Abstract

Phylogeographic studies can resolve relationships between genetic population structure of organisms and geographical distributions. Raccoons have become feral in Japan, and in Hokkaido island, they have been rapidly increasing in number and spreading since the 1970s. We analyzed mitochondrial (mtDNA) and microsatellite DNA to understand the current phylogenetic distribution and invasive founder events. Overall, Hokkaido raccoons maintained high genetic diversity (i.e., the level of heterozygosity was comparable to the original habitat, North America). Based on mtDNA distribution and microsatellite diversity, Hokkaido raccoons were divided into six management units. However, mtDNA haplotype distributions and genetic structures based on microsatellites did not always correspond to each other (e.g., two geographically and genetically separated populations showed similar mtDNA distributions). In addition, a high degree of genetic admixture was observed in every unit, and the degree of genetic differentiation was low even between regions separated by long distances. Compared with other countries in Europe where genetic distribution of introduced raccoons is more clearly structured, the current results represent a unique and complex phenomenon of pet escape/abandonment in Hokkaido: i.e., genetically related colonies were introduced into multiple regions as founder events, resulting in the current state in which raccoons are not clearly genetically differentiated even 40 years after introduction.

## Introduction

Phylogeographic studies can identify relationships between genetic population structures of organisms and geographical habitats^1^. Landscape genetic studies have been conducted on numerous invasive species, from plants to mammals, to find the origin of the population, spread patterns and invasion pathways, and genetic structure and level of genetic variation following invasion^2^.

The raccoon (*Procyon lotor*) is a mammal indigenous to North and Central America^3^ and has become feral in Japan as a result of escape from captivity and unexpected releases since the first report of raccoon naturalization in Inuyama, Aichi Prefecture in 1962^4^. In Hokkaido Prefecture, the northernmost and the second largest island (83,450 km^2^) in Japan, escapes and releases of pet raccoons have been reported since the 1970s, and feral raccoons have been increasing in number and spreading throughout Hokkaido^5,6^. Feral raccoons were initially distributed only in limited regions: 14 and 18 out of 212 municipalities in 1992 and 1995, respectively^6^. However, by 2018, raccoons had been detected in 156 out of 179 municipalities (87.2%) in Hokkaido (Hokkaido governmental report (http://www.pref.hokkaido.lg.jp/ks/skn/alien/araiguma/araiguma_top.htm).

This rapid increase and spreading of raccoons are thought to be due to their biological properties, such as omnivorous behavior, adaptation to cold weather, absence of predators, and high reproductive potential^6^. In Japan, Invasive Alien Species Act was implemented in 2005 by the Ministry of the Environment (MOE) to ensure biodiversity conservation in ecology, to maintain biological safety of human lives and health, and to contribute to the development of agriculture, forestry, and fishery industries. Under the Invasive Alien Species Act, many control programs have been conducted by MOE, the government of Hokkaido, individual municipality and agricultural cooperative societies. To efficiently decrease the raccoon population size, it is necessary to gather basic biological information. Particularly, to infer their dispersion and to reveal population units with geographical or genetic barriers could be useful for effective management^7^.

In many studies, mitochondrial DNA (mtDNA), which has a high mutation rate^8^, has been used as a genetic marker for determining relationships of recent genetic divergence among/within species^9–11^. In particular, the control region (D-loop), which is one of the most variable regions in mtDNA with high substitution rates, has been used in studies of various invasive species^12^. Additionally, using microsatellite analysis, more detailed information can be obtained, such as population structure and within-population diversity, genetic intercrossing, and estimation of the number of founder individuals reported as invasive species, such as raccoons in Europe^13^, Indian mongoose (*Herpestes auropunctatus*) in Okinawa^14^, and sika deer (*Cervus nippon*) in Scotland^15^.

The mtDNA phylogenetic diversity of raccoons has been reported in native habitats (i.e., North and Central America). Pons *et al*.^16^ first detected nine haplotypes and subsequently, Helgen *et al*.^17^ found another four haplotypes in North America. In the same year, Cullingham *et al*.^18^ conducted a phylogenetic study with samples from a wide range of eastern North America and reported 76 haplotypes. Microsatellite analyses have also been reported in native raccoon habitats and revealed population structures in order to utilize the information for zoonosis controls such as rabies^19–21^. Recently, some studies of invasive raccoons in Europe detected the same haplotypes^13,22^ described by Cullingham *et al*.^18^ in the United States. Microsatellite analysis has also shown that multiple introductions of this species happened in Europe^13,22–24^. In Germany, where the oldest introduction event occurred in the 1930s, genetic analysis revealed that at least four independent introduction events resulted in the current genetically-differentiated subpopulations, despite the common assumptions that they stemmed from two separate founding events^24,25^. Microsatellite results in Germany^21,22^, complementary with the mtDNA clusters, also indicated that some founder populations are still genetically distinct 70 years after the last major introduction event.

In Japan, Takada^26^ found 18 mtDNA haplotypes. Takada-Matsuzaki *et al*.^27^ and Takada^26^ investigated phylogenetic distributions in some municipalities (limited to central Hokkaido) and found six mtDNA haplotypes. However, neither dispersal processes based on microsatellite analysis nor comparison of polymorphisms with raccoons in North America has ever been investigated in Japan. Raccoons spread throughout Hokkaido only about 40 years after first being introduced, despite the attempt to control invasion of raccoons under the Invasive Alien Species Act of 2005. Invasion via overland routes from surrounding islands or continents is not possible and human-induced transference and release of raccoons is not likely, as it has been illegal since 2005. Therefore, the present phylogenetic distribution can be a model to describe how past introductions will appear in phylogeographic patterns over time. Thus, the aim of this study is to understand the founder invasion events of raccoons in wider regions of Hokkaido by identifying geographical differentiation via mtDNA and microsatellite analysis.

## Results

### Mitochondrial diversity of raccoons in Hokkaido and other regions

**Figure 1:**
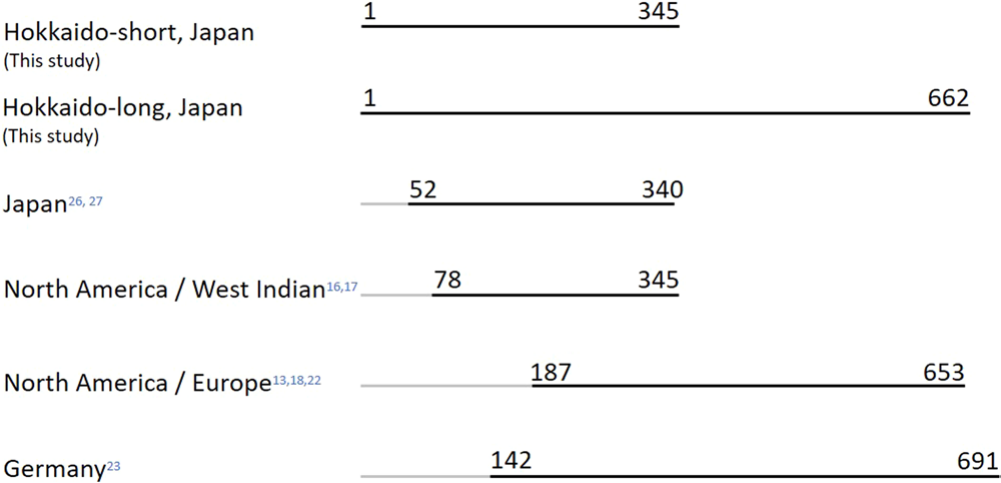
Corresponding mtDNA D-loop fragments investigated in raccoons. Numbers correspond to positions of start/end bases compared with the 662-bp fragment in this study.

With 392-bp fragment, seven haplotypes were detected with 17 polymorphic sites (Table 1): NA (n = 234), HT (n = 117), KK (n = 92), AS (n = 14), AB (n = 62), AH (n = 5), and IW (n = 2) in 526 raccoons collected from 44 municipalities in ten subprefectures in Hokkaido. In a longer 709-bp fragment, an additional nine polymorphic sites were detected, and every sample from the same haplotype showed an identical sequence. Nucleotide sequence data are available in the DNA Data Bank of Japan database as well as GenBank with the following accession numbers: IW: LC455749, HT: LC455750, KK: LC455748, AS: LC455753, NA: LC455747, AB: LC455752, and AH: LC455751. Some haplotypes detected in this study were identified in reports from Japan^26,27^ and North America^18^ (Table 1), though the fragment length is different among the studies (Fig. 1). Polymorphic sites among haplotypes IW, HT, and KK were detected only in base positions 58 and 160, which were not examined in Cullingham’s study^18^. Therefore, these three haplotypes were shown to be the same as PLO32 in North America (Table 1).

**Table 1:** Sequence results of seven haplotypes at different base positions.

	Base position of polymorphic sites																										Haplotype in other studies	
**Haplo type**	0	1	1	1	1	1	1	1	2	2	2	2	2	2	2	2	3	3	3	3	4	4	5	5	6	6	**Japan**^26,27^	**North America**^18^
	5	1	4	5	6	7	9	9	2	6	6	8	8	8	8	9	3	7	7	9	0	2	5	6	5	6		
	8	2	2	7	0	0	7	8	7	0	9	0	1	4	7	2	4	3	4	2	4	3	1	7	8	1		
IW	T	G	A	C	G	T	C	T	A	A	C	A	G	C	T	T	T	A	A	A	C	T	G	G	T	T	RMT 07	PLO 32
HT	C	·	·	·	A	·	·	·	·	·	·	·	·	·	·	·	·	·	·	·	·	·	·	·	·	·	RMT 05	PLO 32
KK	·	·	·	·	A	·	·	·	·	·	·	·	·	·	·	·	·	·	·	·	·	·	·	·	·	·	RMT 02	PLO 32
AS	·	·	·	·	A	·	T	·	·	·	·	·	·	·	C	C	·	·	·	·	·	·	·	·	·	C	RMT 04	ND
NA	·	·	G	·	A	·	·	·	·	G	T	G	A	·	·	C	·	G	·	·	T	·	·	A	·	C	RMT 03	PLO 2
AB	·	A	·	T	A	C	·	C	·	·	·	G	A	T	·	C	C	·	G	G	·	C	T	·	C	·	ND	ND
AH	·	A	·	T	A	C	·	C	G	·	·	G	A	T	·	C	C	·	G	G	·	C	T	·	C	·	ND	ND

Corresponding haplotype IDs identified in the D-loop fragment in this study, reports in Japan by Takada-Matsuzaki *et al*.^27^ and Takada^26^, and a report in North America by Cullingham *et al*.^18^.

**Figure 2:**
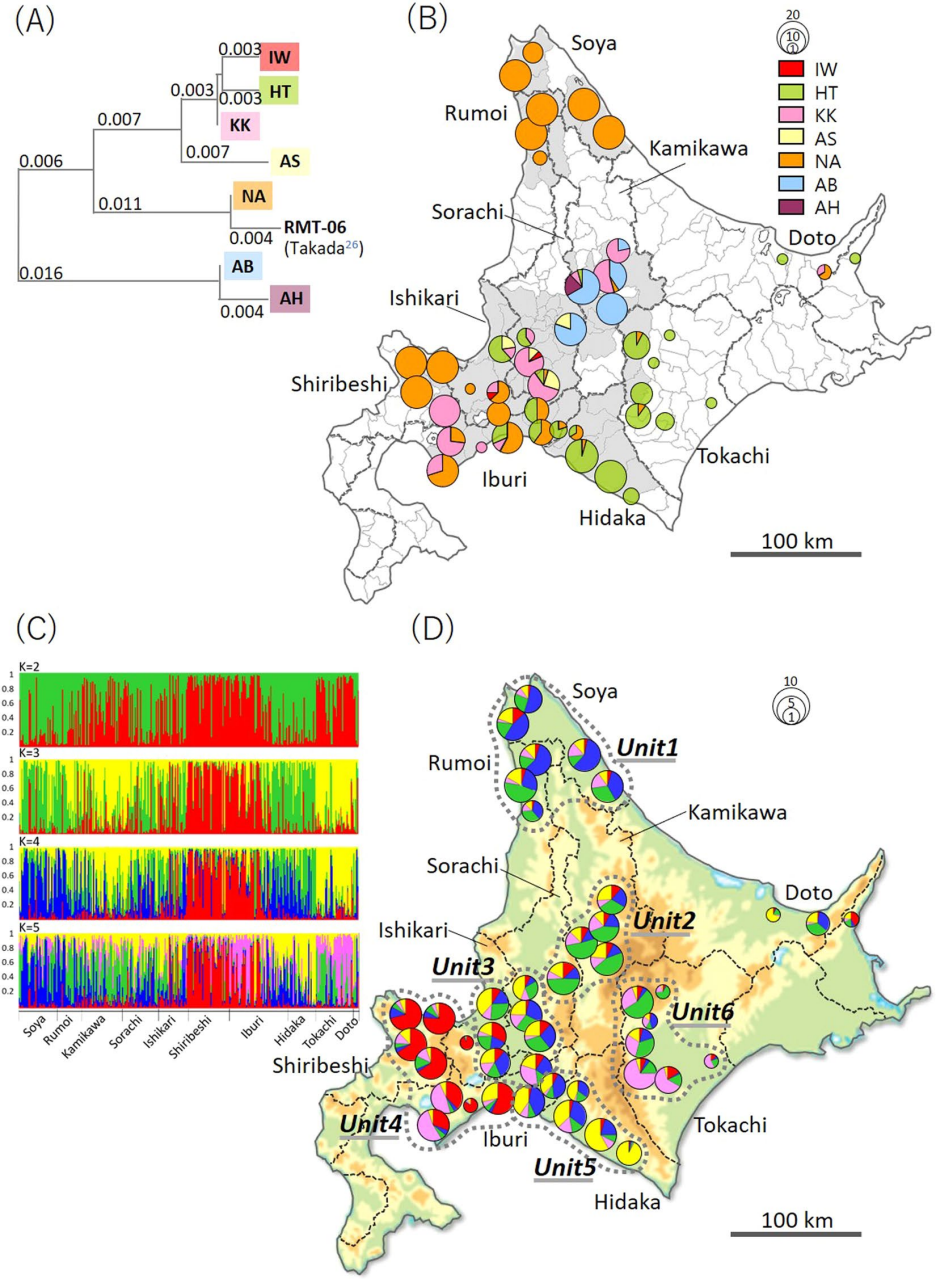
2 A: Neighbor-joining phylogenetic tree of the genetic distances determined by the maximum likelihood method among seven mtDNA haplotypes investigated in this study and RMT-06 reported in Takada^26^. 2B: Geographic distribution of seven mtDNA haplotypes of raccoons (n = 526) in Hokkaido, Japan^52^. Gray areas are municipalities where raccoons were captured in 2009. 2 C: Summary of clustering analysis in STRUCTURE (K = 2–5). Each individual is represented by a single vertical line, representing the estimated population of the individual assignment to the genetic cluster. 2D: Geographic distribution of the STRUCTURE clusters (K = 5) of raccoons (n = 326) in Hokkaido, Japan. The maps in 2B and 2D were created by using Microsoft Power Point office 365 based on the Digital Map published by Geospatial Information Authority of Japan website (https://maps.gsi.go.jp/)^53^.

Haplotype NA was found in almost all subprefectures in the sampling area (Fig. 2B) and detected in about half of the samples (44.5%) investigated in this study. All of the individuals in northern Hokkaido were identified as this haplotype. Haplotype HT was found in about half of the municipalities but mainly in southern-central Hokkaido (Fig. 2B). The majority (95%) of individuals in Tokachi subprefecture were classified as this haplotype. Haplotype KK was found mainly in west-central Hokkaido (Fig. 2B). Haplotypes AS, IW, and AB were found only in limited municipalities (Fig. 2B). Haplotype AH was found only in five individuals in a municipality of Kamikawa Subprefecture (Fig. 2B). This haplotype differed by one nucleotide in base position 227 compared with haplotype AB. The largest number of haplotypes were detected in Kamikawa and the east Sorachi area (six haplotypes), with the second largest in Ishikari and the southwest Sorachi area (five haplotypes).

### Locus-specific diversity in microsatellite analysis

A total of 326 raccoons, which were randomly chosen with maximum 10 samples from each 44 municipality, were genotyped at ten microsatellite loci. The number of alleles per locus ranged from 6 to 19. Single locus heterozygosities observed (H_o_) ranged from 0.66 to 0.87, with 0.75 on average (0.07 standard deviation (SD)). The expected heterozygosities (H_e_) ranged from 0.65 to 0.89, with 0.78 on average (0.08 SD) (Table 2). HWE tests and LD tests were conducted using the Markov chain method (1000 dememorization steps, 1000 batches, and 1000 iterations per batch). Significant values in HWE were different for one locus from five subprefectures (P < 0.05) but were from only two subprefectures following a strict Bonferroni correction for multiple tests (P = 0.05/10 = 0.005); only one locus deviated from HWE in the two subpopulations. Out of 45 pairs of loci in LD tests, one to three pairs were significantly linked by Bonferroni correction in three subprefectures (P = 0.05/45 = 0.0011). However, there were no loci among any pairs that were consistently significant over past studies^18,28–30^, and no value was significant beyond several populations in this study. There was no consistent evidence for LD or null alleles across any populations. Therefore, all loci were included in further analyses.

**Table 2:** Locus-specific diversity measures at ten microsatellite loci for 326 raccoons in Hokkaido, Japan, and other reports from native raccoon habitat in North America.

	Hokkaido (n = 326)			Chicago (n = 323)^28^			Illinois (n = 99)^33^		
	Na	Ho	He	Na	Ho	He	Na	Ho	He
**PLO3–71**	9	0.75	0.79	—	—	—	12	0.69	0.77
**PLO-M20**	12	0.83	0.86	15	0.83	0.82	13	0.83	0.87
**PLO2–14**	18	0.74	0.80	29	0.83	0.82	20	0.83	0.88
**PLO-M3**	6	0.70	0.68	9	0.76	0.79	7	0.85	0.79
**PLO-M2**	11	0.76	0.83	17	0.81	0.84	14	0.84	0.87
**PFL11**	13	0.87	0.87	19	0.84	0.84	16	0.87	0.89
**P140**	7	0.66	0.65	12	0.73	0.73	9	0.72	0.74
**PFL9**	9	0.71	0.73	13	0.77	0.79	11	0.83	0.83
**PLO-M17**	8	0.68	0.72	11	0.81	0.75	8	0.76	0.79
**PLO3–86**	19	0.86	0.89	37	0.75	0.88	24	0.94	0.91

The number of alleles (Na), observed heterozygosity (Ho), expected heterozygosity (He).

The data set from Chicago was reported in Santonastaso *et al*.^28^.

The data set from Illinois was reported in Hauver *et al*.^33^.

### Population structure and genetic diversity

Structure Harvester results analyzed based on 44 municipalities showed the lowest *ΔK* when *K* = 4 (Supplement file 1). However, log-likelihoods were not high at *K* = 4 and increased beyond *K* = 4. The highest values were obtained for *K* = 5, and, therefore, we chose *K* = 5 as an appropriate number of clusters. In the STRUCURE analysis, 44 municipalities were divided into five clusters, which consisted of the following subprefectures: 1: Soya and Rumoi; 2: Kamikawa, Sorachi, and Ishikari; 3: Shiribeshi and Iburi; 4: Hidaka; 5: Tokachi and Doto (Fig. 2C). However, mtDNA distributions in the area of Kamikawa and east Sorachi were distinct from those in the area of southwest Sorachi and Ishikari. Therefore, based on the mtDNA distributions and microsatellite diversity with *K* = 5, we divided raccoon groups into six management units in Hokkaido (Fig. 2D), as follows: Unit1 (Soya and Rumoi subprefectures), Unit2 (Kamikawa and east Sorachi subprefectures), Unit3 (the region covering southwest Sorachi, Ishikari, and a part of Iburi subprefectures), Unit4 (Shiribeshi, west Iburi, and west Ishikari subprefectures), Unit5 (Hidaka and east Iburi subprefectures), and Unit6 (Tokachi Subprefecture). With 321 raccoons forming these six management units, H_o_ ranged from 0.726 to 0.784 with an average of 0.75 (0.02 SD). H_e_ ranged from 0.731 to 0.787 with an average of 0.761 (0.02 SD) (Table 3). Pairwise F_ST_ values among the six units were statistically differentiated between any two units, and values were low, ranging between 0.0045 to 0.0667 (Table 4).

**Table 3:** Genetic diversity in six management units for 321 raccoons in Hokkaido, Japan.

	Number of samples	Average value of allelic richness	H_o_	H_e_	F_IS_
**Unit1**	60	7.406	0.768	0.7592	−0.012
**Unit2**	49	8.054	0.784	0.7799	−0.005
**Unit3**	62	8.617	0.766	0.7874	0.027
**Unit4**	72	7.692	0.740	0.7741	0.044
**Unit5**	42	7.280	0.726	0.7310	0.007
**Unit6**	36	7.000	0.736	0.7319	-0.006
**ALL**	321	8.803	0.755	0.7833	

Average values of allelic richness (Ar), observed heterozygosity (H_o_), expected heterozygosity (H_e_), fixation index (F_IS_).

**Table 4:** Pairwise F_ST_ values among the six management units detected in Hokkaido, Japan.

	Unit1	Unit2	Unit3	Unit4	Unit5
**Unit2**	0.0247				
**Unit3**	0.0102	0.0045			
**Unit4**	0.0406	0.0245	0.0177		
**Unit5**	0.0360	0.0435	0.0250	0.0437	
**Unit6**	0.0459	0.0217	0.0286	0.0374	0.0667

## Discussion

In the present study, mitochondrial and nuclear diversity was examined in feral raccoons introduced into Hokkaido, Japan. This is the first report to study raccoon population dynamics based on genetic differentiation in Japan and to compare genetic characteristics among this animal in Japan with an original habitat in North America.

The 662-bp sequences detected in this study covered fragments identified in previous studies, including 289 bp in Japan^27^, 268 bp in North America and West Indian islands^16,17^, and also 467 bp in eastern North America^18^ (Fig. 1). The sequences also partially covered 521 bp out of a 550 bp fragment from Germany^23^. Here, seven haplotypes were detected from 526 samples. Five of these were reported previously in central Hokkaido^27^. When adding RMT06, which had been found only in the study by Takada-Matsuzaki *et al*.^27^ but not in the present study, founder events in Hokkaido were established from at least eight female haplotypes (Fig. 2A). This could be an underestimation, given that founder phylogenetic diversity can diminish during changing demographic periods based on the number of founder females and survivorship. Compared with reports in native habitats in Northeastern America, where 76 haplotypes were detected from 311 samples^18^, the number of haplotypes in Hokkaido is small. Such low mitochondrial diversity was also reported in other introduced areas in Europe, with two haplotypes identified from 58 samples in Spain^13^, six haplotypes from 193 samples in Germany^23^, and four haplotypes from 72 samples from the border area in Poland, Czech Republic, and Germany^22^. Rapid expansion from small populations can involve serial bottlenecking with progressive loss of allelic diversity, so that populations in more recently colonized locations could contain less genetic diversity^31^.

Four haplotypes out of eight identified in this study were also detected in North America^18^ and five haplotypes out of the eight were also detected in the other locations within Japan^32^, indicating that raccoons from the same origin group in North America were introduced at several remote places in Japan as founder events. Another possibility is that re-introduction (as a second release in the prefectures) from the initial introduced regions into the non-habitat area, happened after the first founder event as discussed by Takada-Matsuzaki *et al*.^27^ Haplotype NA matched haplotype PLO2, which was mentioned in Cullingham’s study^18^. PLO2 is the most common raccoon haplotype, is widespread in North America, and has also been reported in non-native areas, such as Germany, Spain, Poland, and Czech Republic^13,22,23^. Haplotype NA was detected in introduced areas across countries, which strongly suggests that the same genetic population of this haplotype was managed under captivity from wild populations in North America, exported as pets or as a fur-bearing animal and was introduced into non-native regions.

In this study, 326 individuals were divided into six groups based on mtDNA polymorphism, STRUCTURE analysis, and habitat regions, by use of the municipality as a unit. We note that this classification might not be biologically relevant for wild animals that move regardless of the frontiers between municipalities. In Hokkaido, however, most eradication programs of raccoons have been implemented by each municipality. Therefore, this study focused more on classifying management units, rather than on finding the ecological boundary between sub-populations.

Genetic diversity at these ten microsatellite loci in Hokkaido raccoons is low in N*a* though it was at the same level in H_o_ and H_e_ when compared to that in a native habitat of North America (in Chicago^28,33^ and Illinois^34^) (Table 2). When genetic variability defined by clusters was evaluated in the six management units (Table 3), Ar, H_o,_ and H_e_ were high compared to Germany where feral raccoons are widespread throughout the country^24^. Values were low in six clusters of 407 feral German raccoons: Ar: 3.6–4.9, H_o_: 0.52–0.64, and H_e_: 0.53–0.65. These differences demonstrate that nuclear genetic diversity in Hokkaido has stayed polymorphic at the same level as native habitat, in contrast to other invaded habitats.

In other raccoon-invaded areas, genetic diversity at the nuclear level in Spain was lower than in Missouri, a native area^13^. Raccoons were first reported in Spain relatively recently, in 2003. The microsatellite analysis consisted of 58 samples and showed only two mtDNA haplotypes captured in the limited area of Madrid. Therefore, diversity may be lower than observed in our study and in reports from the United States. In contrast, raccoons have been in Germany much longer than in Spain. There, raccoons were introduced in the 1930s, but the populations were derived from two separate founding events and one additional escape event^24^. These small numbers of founding events likely led to low polymorphisms seen in Germany^24^. The high genetic polymorphisms observed in this study indicate that the founder event in Hokkaido was relatively complex compared to that in Spain or Germany. The mtDNA of raccoons in Hokkaido showed allopatric, whereas the genome DNA showed very low pairwise F_ST_ values between any two management units regardless of the distance of the two units. Almost all the F_ST_ values (0.005–0.067) in the current study was lower than the lowest value observed in Germany (0.049)^23^. This indicates that several maternal populations, with different genetic backgrounds, were introduced into different regions in Hokkaido. However, these original populations were closely related to each other, resulting in limited differentiation among Units even 40 years after raccoons were introduced.

In Japan, raccoons were mainly imported as pets and introduced into the wild afterwards^35^. To our best knowledge, there are no documents recording when and how many raccoons were imported into Japan^7^, though the results of this study reflect that founder populations in Hokkaido likely derived from genetically related individuals from a captive breeder in North America or the main island of Japan. When allelic richness was compared among the six management units in Hokkaido, Unit2 and Unit3 showed relatively high genetic diversity and also highly diverse mtDNA (Table 3). Unit3, covering Sorachi, Ishikari, and Iburi subprefectures (the most human populated regions), which includes Sapporo, the largest city in Hokkaido. Unit2 has the second largest city, Asahikawa, suggesting that raccoons from multiple haplotypes were released into highly populated regions. Historical evidence demonstrates that Ishikari, Sorachi, and Kamikawa subprefectures were regions where feral raccoons or their traces were detected in the 1990s, earlier than in other regions (Hokkaido governmental reports and Ikeda^7^). A report showing that national parks with high surrounding human population density had significantly more alien species^36^ also supports our findings. Additionally, in Hokkaido, genetic polymorphism was varied but conserved, not only in highly populated areas, but also in regions throughout the island. This result suggests that several founder females with different haplotypes were introduced sympatrically and/or allopatrically, and cross-municipality migrating has resulted in the genetic diversity observed in Hokkaido.

Ikeda^6^ reported that the naturalization of raccoons in Hokkaido was due to multiple escapes and abandonment of pets. They also reported that additional human-induced transport and secondary releases have allowed habitat expansion in more remote locations within Hokkaido. Raccoons tend to disperse along the rivers and water passages^37^. Females are often philopatric, while males are more likely to disperse^37,38^. In the native habitat, the majority of raccoons relocate less than 5 km^39^, meanwhile they sometimes disperse more than 20 km in the absence of major landscape barriers^30^. Genetic analysis here suggests that geographical barriers prevent migration of feral individuals and reduce genetic admixture between some populations. The mtDNA haplotype of Unit1 and a part of Unit4 were the same; genetic structure in microsatellite clusters was different between these two units, indicating that there was less genetic intercrossing between north and southwest regions in Hokkaido. Geographical distances between these regions also support that there has been less genetic admixture between these units after individuals were introduced from the same origin. Unit2 was limited to Kamikawa and Sorachi Subprefectures, and Unit6 in Tokachi Subprefecture, which are neighboring regions. Although mtDNA haplotypes were different between these two units, STRUCTURE analysis inferred possible genetic admixture, suggesting that male individuals might have been dispersed among these regions.

Unit5 in Hidaka Subprefecture and Unit6 are also neighboring regions and have the same mtDNA haplotype constitution. However, STRUCTURE results indicated that there was less individual contact. There is approximately 150 km of the Hidaka mountain range between Hidaka and Tokachi, which could have prevented genetic mixture by serving as a geographical barrier, resulting in each population having maintained their own genetic structure. It is thus important for neighboring areas to cooperate with each other to control the spread of invasive raccoons. In the case of Hidaka and Tokachi, there could be effective management considering the geographical barriers^19^. Although human-induced re-introduction and transport were reported at the beginning of raccoon naturalization, the Invasive Alien Species Act of 2005 strictly regulates transportation of living animals. In this situation, biological information, such as geographical isolation or populational genetics, will be helpful for effective eradication and control^19^.

## Summary

1. In Hokkaido, founder events of raccoon introduction occurred sympatrically and allopatrically in separate regions, which reflects a unique phenomenon of pet escape/abandonment with different maternal populations (consisting of at least eight mtDNA haplotypes) that were closely related with each other.
2. Genetic diversity of raccoon has stayed polymorphic at almost same level as native habitat, and a high degree of genetic admixture was detected in regions where humans were highly populated.
3. Some populations have maintained their own genetic structure, in areas of geographical barriers.

Compared with other countries in Europe where genetic distribution is more clearly structured in introduced raccoons, the current results show that genetically related colonies were introduced into multiple regions as founder events, resulting in the current state where raccoons are not clearly genetically differentiated even 40 years after introduction.

## Materials and Methods

### Sample collection and DNA extraction

Hair, whisker, or muscle samples were collected from 526 carcasses of feral raccoons that were euthanized for eradication control in 44 municipalities in the main island of Hokkaido from 2010 to 2013. The sampling area was chosen based on where raccoons were captured in 2009 (based on the data from the government of Hokkaido, gray areas in Fig. 2B). Hair and whisker samples were kept at −20 °C with silica gel until DNA extraction. DNA was extracted to final volumes of 30 to 50 µL using an ISOHAIR Kit (Nippon Gene, Tokyo, Japan) according to the manufacturer’s instructions. Muscle samples were cut into small pieces and kept in 99% ethanol at −20 °C until DNA extraction. DNA was extracted to final volumes of 50 µL using a Wizard Genomic DNA Purification Kit (Promega, Madison, WI, USA) according to the manufacturer’s instructions.

### mtDNA PCR amplification and sequence analysis

Amplification was performed for 526 samples using the universal primers described by Helgen *et al*.^17^, Proc L (5′-TCATCGAAAATAATCTGTTAAAATGAA-3′) and Proc H (5′-CGGAGCGAGAAGAGGTACAC-3′), which amplify a 392-bp fragment of the mitochondrial D-loop region of raccoon mtDNA. For PCR, a 25-µL reaction volume was used, including 1 µL DNA extract, 0.625 U of KAPATaq EXtra DNA polymerase (NIPPON Genetics Co. Ltd., Tokyo, Japan), 5 µL of 5× KAPATaq EXtra buffer, 5 µL of 25 mM MgCl_2_, 0.75 µL of dNTP mix (10 µM each), and 1.25 µL of each of the primers (final concentration of 0.5 µM) described above. Reaction conditions were as follows: 1 cycle at 95 °C for 10 min; 35 cycles of denaturation at 95 °C for 30 s, annealing at 53 °C for 30 s, and extension at 72 °C for 30 s; and 1 cycle at 72 °C for 10 min. To confirm amplification, 5 µL of the products were electrophoresed on a 1.5% agarose gel. The products were purified using a FastGene Gel/PCR Extraction Kit (NIPPON Genetics Co., Ltd.). The products were sequenced using a Big Dye Terminator v1.1 Cycle Sequencing Kit (Applied Biosystems, Foster City, CA, USA). The sequences were analyzed using an ABI PRISM 310 Genetic Analyzer (Applied Biosystems) and differentiated into several haplotypes. Subsequently, to compare these 392-bp sequences in Hokkaido with those in North America^18^, another reverse primer, ProcR-add (5′-CCATGAATTAAACTGCACCA-3′), was designed for amplification of longer 709-bp fragments based on the sequence reported by Frantz *et al*.^23^. In 46 samples, which were picked from seven haplotype groups, covering all detected subprefectures, longer 709-bp fragments were amplified with primers Proc L and ProcR-add and sequenced as described above, in order to confirm that each sequence detected in Hokkaido was identical to the haplotype reported in North America (Fig. 1).

### Molecular data analysis

Seven haplotypes of the D-loop fragment sequence and one haplotype reported in a previous study^26^ were aligned and a neighbor-joining phylogenic tree was constructed using MEGA5 software (http://www.megasoftware.net/)^40^. Genetic distances, based on alignment gaps among the haplotypes, were estimated from all substitutions and by using the two-parameter distance method^41^.

### Microsatellite genotyping

A total of 326 individuals from all 44 municipalities were genotyped at 10 microsatellite loci (Table 5). From municipalities with>10 samples, 10 samples were randomly selected. From the other municipalities, with 10 or less samples, all samples were used for the microsatellite analysis. Primers and primer combinations used in multiplex PCR assays are shown in Table 5. Multiplex PCR reactions were performed in a total volume of 15 µL, consisting of 1 µL of DNA solution, 0.075 µL of Kit Mix 1 (Multiplex Assay Kit; Takara Bio Inc., Shiga, Japan), 7.5 µL of Kit Mix 2, 0.5 µL of primer mix (0.25 µM each), and 5.925 µL of PCR-grade water. The mixture was heated to 94 °C for 1 min, followed by 40 cycles of 30 s at 94 °C, 1 min at 52–56 °C, and 1 min at 72 °C, with final 10 min at 72 °C. One microliter of product was diluted in 120 µL of distilled water, and 1 µL of this diluted solution was mixed with 0.125 µL of GeneScan 500 LIZ size standard (Life Technologies Japan Ltd., Tokyo, Japan) and 10 µL of Hi-Di formamide (Life Technologies Japan Ltd.). After denaturation at 95 °C for 3 min and cooling on ice, each sample was analyzed with the ABI PRISM 310 Genetic Analyzer (Life Technologies Japan Ltd.). Allele size was determined using GeneScan ver. 4.1 software (Life Technologies Japan Ltd.). When PCR amplification was weak for any locus, a single PCR reaction was performed with the same primers.

**Table 5:** Primer sequences for microsatellite analysis.

Locus	Mult. PCR	Dye	F-primer sequences (5′-3′)	Primer conc.	Ta	References
			R-primer sequences (5′-3′)			
PLO3–71	A	PET	GCTTCCTTTAATTTTAACTAATTG^§^	0.35	56	^49^
			CAATCCTGTATCAGGTTTCC			
PLO-M20	A	VIC	GATTCTTATGTCTCTTGGGA	0.15	56	^49^
			AAGTGCTTCAAGAGAAGTGC^§^			
PLO-M3	A	NED	CTCCCATCTTCCTCTTTTCG	0.1	56	^49^
			GTTGACAATTGCAGGACCAC			
PLO2–14	A	FAM	AAGAGCGTAATAAAAGCTTAC	0.35	56	^49^
			CAAATAACAAGTTTCAATTTGG			
PLO-M2	A	PET	GGAAAACCACAGAGAGACGG	0.3	56	^49^
			CTTGGCACAGAGCAGAATCC			
PFL11	B	PET	CATGCAAATAACACGCAC	0.4	52	^50^
			CTGAACAAGGTAGGAAAGTCACTC			
P140	B	FAM	ACCAGGCAATGGTAATACAG	0.15	52	^51^
			CCAGGAGGACTTGTCAGAT			
PFL9	B	VIC	GCCTTCATTTAGTTGAGGTCAG	0.2	52	^50^
			GCATTCTGTCAGTGGCTTTCAC			
PLO-M17	B	NED	CTGCTGAGTAAGGAGTAAGG	0.35	52	^49^
			TCCCCTGTACATATTCAGGC			
PLO3–86	B	FAM	GATTGATAGATTAATTGGTCTTAACTTCC	0.27	52	^49^
			CTGGATTATAAATCTGGCAAGAGCC			

^§^Original primer sequences were modified based on GenBank #DQ388436.1.1 and DQ388437.1.

### Data analysis

The software CERVUS 3.0.7^42^ was used to calculate loci characteristics, including number of alleles (Na), observed and expected heterozygosities (H_o_ and H_e_), and polymorphic information content. In addition, we tested whether there was any evidence suggesting a departure from Hardy-Weinberg equilibrium (HWE) of ten microsatellite loci and linkage disequilibrium (LD) between populations using the web-based program GENEPOP 4.2^43,44^.

Bayesian clustering and assignment analyses were performed with STRUCTURE 2.3.4^45^ for 44 municipalities including 326 samples in ten subprefectures (i.e., Soya, Rumoi, Kamikawa, Sorachi, Ishikari, Shiribeshi, Iburi, Hidaka, Tokachi, and Doto; Fig. 2). The Structure Harvester^46,47^ analysis was conducted with 10 repetitions of 100,000 interactions of Markov chain Monte Carlo, following a burn-in of 100,000 interactions at *K* = 12. Estimation of the most probable number of subpopulations was based on the log-likelihood values associated with each *K*.

### Genetic structure and data analysis

The following analysis was done in six management units, estimated based on the combination of mtDNA distributions and STRUCTURE analysis (described above). Among 326 samples used in the STRUCTRE analysis, five samples from the Doto region were excluded from the analyses, due to the limited number of samples (i.e., 321 samples in total). Allele richness (Ar) and within-population inbreeding coefficient (F_IS_) values for each management unit were calculated by FSTAT 2.9.4^48^. Pairwise genetic differentiation coefficient (Fst) values among management units were calculated using GENEPOP 4.2.

## Supplementary information

Supplementary information.
