# Supplementary material for: Genetic population structure of invasive raccoons (Procyon lotor) in Hokkaido, Japan: Unique phenomenon caused by pet escape or abandonment
Source: Sci Rep. 2020 May 15;10:8108. doi: 10.1038/s41598-020-64526-y (PMC7229193; doi:10.1038/s41598-020-64526-y)

Title Page

Title

Genetic population structure of invasive raccoons (*Procyon lotor*) in Hokkaido, Japan: Unique phenomenon caused by pet escape or abandonment

The names of the Authors

Minami W OKUYAMA, Michito SHIMOZURU, Mariko NAKAI, Emi YAMAGUCHI, Kei FUJII, Ken-ichiro SHIMADA, Tohru IKEDA, Toshio TSUBOTA

This document produced by the Structure Harvester,
http://taylor0.biology.ucla.edu/structureHarvester
Written by Dent Earl, dearl (a) soe ucsc edu.
CITATION:
Earl, Dent A. and vonHoldt, Bridgett M. (2012)
STRUCTURE HARVESTER: a website and program for visualizing
STRUCTURE output and implementing the Evanno method.
Conservation Genetics Resources vol. 4 (2) pp. 359-361 doi: 10.1007/s12686-011-9548-7
Core version: vA.2 July 2014
Plot version: vA.1 November 2012
Web version: v0.6.94 July 2014
File generated at 2016-Oct-11 16:48:36 PDT

##########

# K	Reps	Mean LnP(K)	Stdev LnP(K)	Ln'(K)	|Ln''(K)|	Delta K
1	10	−11,968.350	0.288	–	–	–
2	10	−11,756.650	1.660	211.700	100.930	60.796
3	10	−11,645.880	4.394	110.770	78.390	17.840
4	10	−11,613.500	7.304	32.380	5.270	0.722
5	10	−11,586.390	25.195	27.110	202.740	8.047
6	10	−11,762.020	141.288	−175.630	183.560	1.299
7	10	−11,754.090	168.201	7.930	136.380	0.811
8	10	−11,882.540	333.798	−128.450	229.590	0.688
9	10	−11,781.400	190.142	101.140	239.510	1.260
10	10	−11,919.770	388.592	−138.370	178.380	0.459
11	10	−11,879.760	140.268	40.010	254.320	1.813
12	10	−12,094.070	259.183	−214.310	–	–

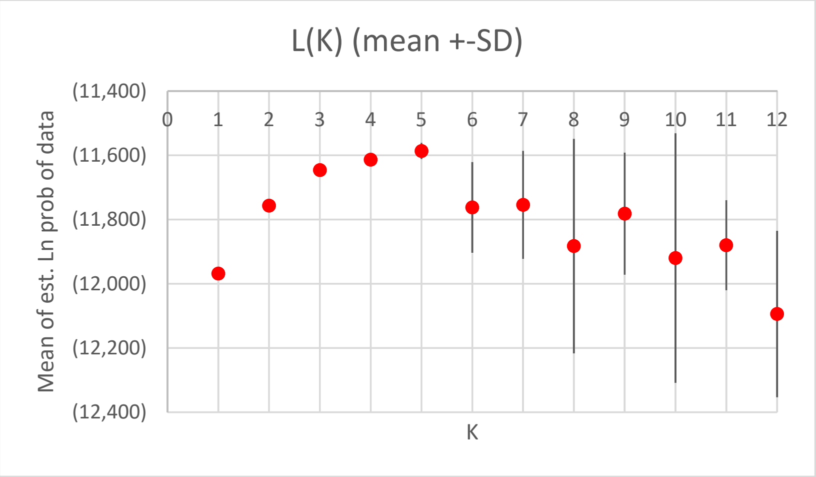

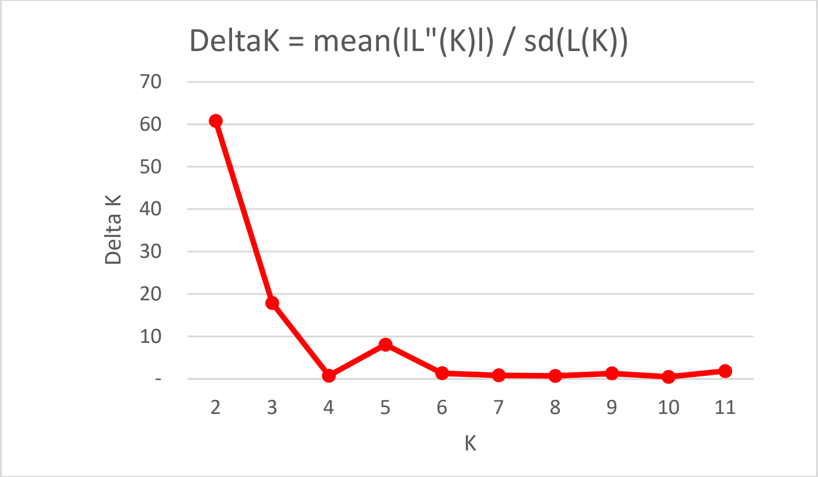

Supplement: Supplementary file 1 — Supplementary information. [file 41598_2020_64526_MOESM1_ESM.pdf]
